# Stromal cells promote anti-estrogen resistance of breast cancer cells through an insulin-like growth factor binding protein 5 (IGFBP5)/B-cell leukemia/lymphoma 3 (Bcl-3) axis

**DOI:** 10.18632/oncotarget.5624

**Published:** 2015-10-19

**Authors:** Benjamin Leyh, Angela Dittmer, Theresia Lange, John W. M. Martens, Jürgen Dittmer

**Affiliations:** ^1^ Clinic for Gynecology, Martin Luther University Halle-Wittenberg, Halle/Saale, Germany; ^2^ Department of Medical Oncology, Erasmus University Medical Center, Rotterdam, The Netherlands

**Keywords:** stromal cells, anti-estrogen resistance, fulvestrant, mesenchymal stem cells, carcinoma-associated fibroblasts

## Abstract

There is strong evidence that stromal cells promote drug resistance of cancer. Here, we show that mesenchymal stem cells (MSCs) and carcinoma-associated fibroblasts (CAFs) desensitize ERα-positive breast cancer cells to the anti-estrogen fulvestrant. In search for the mechanism, we found that MSCs and CAFs similarly increased the activity of the PI3K/AKT and the JAK/STAT3 pathways and upregulated the expression of integrin β1, IGF1R, HIF1α, CAIX and Bcl-3 in MCF-7 cells. Further analyses revealed that MSCs and CAFs coordinately induce these changes by triggering the downregulation of IGFBP5. Loss of IGFBP5 in MCF-7 cells was an early and long-lasting event in response to MSCs and CAFs and was accompanied by growth stimulation both in the absence and presence of fulvestrant. The growth-stimulatory effect in the absence of fulvestrant could be attributed to PI3K/AKT pathway activation and could be mimicked by insulin. The growth-promoting effect in the presence of fulvestrant depended upon the upregulation of Bcl-3. By cRNA microarray analysis we identified additional IGFBP5 targets, of which two (KLHL4 and SEPP1) were inversely regulated by IGFBP5 and Bcl-3. BT474 cells also responded to stromal cells by downregulating IGFBP5 and upregulating the P-AKT, Bcl-3 and IGF1R levels, whereas T47D cells did not show any of these responses. In conclusion, our data suggest that, by targeting IGFBP5 expression in ERα-positive breast cancer cells, such as MCF-7 cells, MSCs and CAFs are able to orchestrate a variety of events, particularly activation of the PI3K/AKT pathway, upregulation of Bcl-3 expression and desensitization to anti-estrogen.

## INTRODUCTION

Breast cancer is the most frequent cancer in women and the leading cause of cancer death in women world-wide [1]. As a heterogeneous disease breast cancer can be divided into subgroups either based on immunochemical or gene expression analysis [2]. Immunochemically, the ERα (estrogen receptor α)-positive breast cancer, the most common breast cancer subtype, can be distinguished from Her2 (human epidermal receptor 2)-positive and triple negative breast cancers (negative for ERα, Her2 and progesterone receptor). ERα-positive breast cancers can selectively be treated with anti-estrogens or aromatase inhibitors (endocrine therapy). SERM (selective estrogen receptor modulator)-like anti-estrogens, such as tamoxifen, act by blocking ERα activity, SERDs (selective estrogen receptor downregulators), such as fulvestrant, additionally downregulate ERα expression. Aromatase inhibitors interfere with ERα activity by inhibiting estrogen synthesis, thereby causing estrogen deficiency. Anti-estrogens or aromatase inhibitors significantly reduce breast cancer mortality of patients suffering from ERα-positive, but not ERα-negative breast cancer confirming the selectivity of these drugs [3]. The success of endocrine therapies, however, is limited by resistance to these drugs (endocrine resistance), either pre-existing (intrinsic resistance) or developed in the course of treatment (acquired resistance) [4, 5].

Acquired resistance can be facilitated by the tumor stroma [6–8]. Both, extracellular matrix and stromal cells are able to protect cancer cells against drugs. The carcinoma-associated fibroblast (CAF), an important component of the tumor stroma and involved in tumor progression, plays a major role in the acquisition of drug resistance [9]. CAFs, a type of activated fibroblasts, can be generated from different cell types, among which is the mesenchymal stem cell (MSC) [10]. MSCs are commonly residing in the bone marrow and are attracted to wounds and cancer lesions [11, 12]. Once having entered a cancer lesion, they start interacting with the tumor cells, which most often leads to cancer progression. Like CAFs, MSCs have found to contribute to the acquisition of drug resistance [8, 13].

There are many mechanisms described that could lead to endocrine resistance [4, 5]. Often, this involves the activation of the survival pathway PI3K (phosphoinositol-3-kinase)/AKT [14]. Also the activation of the Ras/Raf/MEK1/ERK1/2 pathway can be protective against ERα-directed drugs. The activation of either pathway can lead to phosphorylation of ERα, allowing ERα to act independently of estrogen. Both pathways are commonly activated through receptor tyrosine kinases (RTKs) [15]. Of the RTKs, IGF1R (insulin-like growth factor 1 receptor) may be of particular importance for endocrine resistance, as it interacts with ERα [16] and its agonist IGF-1 (insulin-like growth factor 1) shares with ERα the ability to down-regulate critical growth repressor genes [17]. Regulation of IGF1R activity is complex and is not only controlled by its ligands IGF-1 and −2, but also by the IGF inhibitors IGFBP1-6 (IGF binding proteins 1–6) [15]. Interestingly, of these inhibitors, IGFBP2 has been shown to regulate ERα activity [18]. Of note, IGFBPs can also act in an IGF-independent manner [19]. E.g., IGFBP5 has been reported to increase survival of MCF-7 cells in nutrient-poor conditions [20].

Besides RTKs, integrins, such as integrin β1, are typical activators of PI3K/AKT and Ras/Raf/MEK1/ERK1/2 pathways [21] and have also been linked to endocrine resistance [8]. Also the NFκB (nuclear factor of κB) pathway, which interferes with ERα activity in multiple ways [22, 23], has been implicated in the acquisition of endocrine resistance [24, 25]. Specifically, Bcl-3 (B-cell leukemia/lymphoma 3), a member of the atypical IκB family and regulator of NFκB activity in the nucleus [26, 27], has been shown to promote proliferation of MCF-7 cells under estrogen deficiency [28]. Bcl-3 is also linked to breast cancer metastasis [29].

CAFs have been shown to induce resistance to the SERM tamoxifen by activating the PI3K/AKT and/or Ras/Raf/MEK1/ERK1/2 pathways [30, 31], though other mechanisms have also been reported, which include synthesis of estrogen by CAFs [32] or secretion of ketone bodies and lactate by autophagic CAFs [33]. Little is known about the mechanisms by which MSCs induce resistance to ERα-targeting drugs. One report shows that higher expression of the progesterone receptor (PR) may be linked to the ability of MSCs to support growth of ERα-positive MCF-7 breast cancer cells under estrogen deficiency [34].

We explored the possibility that MSCs and CAFs may interfere with the SERD-like anti-estrogen fulvestrant (ICI 182,780), which also downregulates ERα protein levels. We found that either stromal cell type promoted growth of ERα-positive breast cancer cells, such as MCF-7 cells, in the presence of fulvestrant. Searching for the underlying mechanism, we identified Bcl-3 as a major mediator of protection against fulvestrant and found that Bcl-3 was regulated by MSCs and CAFs along with other proteins and phospho-proteins in a coordinated manner through IGFBP5.

## RESULTS

### MSCs and CAFs promote resistance of MCF-7 cells to the anti-estrogen fulvestrant

**Figure 1:**
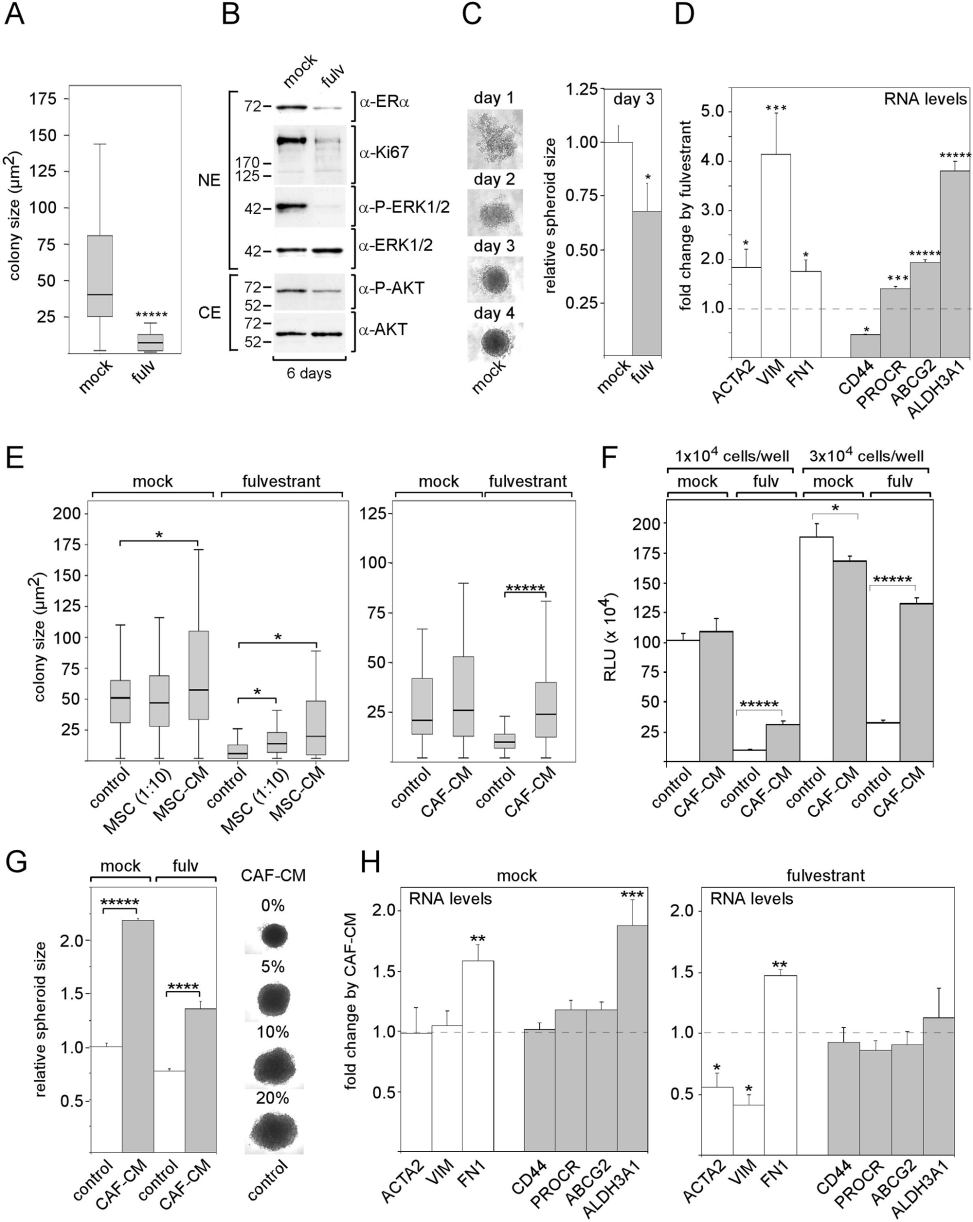
MSCs and CAFs promote growth of MCF-7 cells in the presence of fulvestrant Effect of 100 nM fulvestrant on **A.** colony size of MCF-7 cells in the clonogenic assays, **B.** expression of ERα, Ki67, P-ERK1/2 and P-AKT levels in Western blot analysis, **C.** spheroid formation and **D.** RNA expression of mesenchymal markers (ACTA, VIM, FN1) and stem cell markers (CD44, PROCR, ABCG2, ALDH3A1). **E, F.** Effects of MSCs, MSC-CM and CAF-CM on MCF-7 cell growth in the presence and absence of fulvestrant. Cell growth was either determined by measuring the sizes of individual clones in the clonogenic assay (E) or by the ATP-based growth assay (F). **G, H.** Effect of CAF-CM on (G) spheroid size in the presence and absence of fulvestrant and (H) on the RNA levels of mesenchymal and stem cell markers in the absence and presence of fulvestrant. In (A, E), the data of a representative experiment are shown, in (D, F–H), each bar represents the mean value ± S.D. of at least three independent experiments. Statistical analysis for the clonogenic assay was performed by the Wilcoxon test (A, E). Other statistical analyses were done by using the student's *t*-test. ACTA = α-smooth muscle actin, VIM = vimentin, FN1 = fibronectin-1, ABCG2 = ATP binding cassette subfamily G2), ALDH3A1 (aldehyde dehydrogenase 3 family, member A1), NE/CE = nuclear/cytosolic protein extract, RLU = relative light units.

To show that the anti-estrogen fulvestrant interferes with the activity of MCF-7 breast cancer cells, we examined the effects of fulvestrant on cell growth, on the expression of selected proteins, on spheroid formation and on expression of mesenchymal and stem cell markers. The incubation of MCF-7 cells with fulvestrant at a final concentration of 100 nM for six days strongly reduced growth of individual clones in clonogenic assays (Figure 1A). Along with it, the protein expression of the proliferation marker Ki67 substantially decreased (Figure 1B). Also, as expected, fulvestrant diminished the protein level of ERα (Figure 1B). Furthermore, fulvestrant downregulated P-AKT and P-ERK1/2 levels (Figure 1B) suggesting that fulvestrant exerts an inhibitory effect on the PI3K/AKT- and the Ras/Raf/MEK/ERK1/2 pathways. Fulvestrant also interfered with cell aggregation in 3D suspension cultures and led to the generation of smaller spheroids (Figure 1C). In addition, fulvestrant significantly induced the RNA expression of the mesenchymal proteins vimentin, fibronectin and ACTA2 (α-smooth muscle actin) und induced the expression of a couple of stem cell markers, such as PROCR, ABCG2 (ATP binding cassette subfamily G2) and ALDH3A1 (aldehyde dehydrogenase 3 family, member A1) (Figure 1D) while reducing the expression of the stem cell marker CD44. This may suggest that fulvestrant promotes the expansion of a pool of cells of a more mesenchymal phenotype, which may be more resistant to fulvestrant.

Next, we studied the effects of MSCs and CAFs on MCF-7 growth in the presence and absence of fulvestrant. When MCF-7 cells were co-cultured with MSCs at a ratio of 10:1 for five days and compared to MCF-7 cells grown alone, average colony size was significantly increased in the presence, but not in the absence of fulvestrant (Figure 1E). Growing MCF-7 cells in growth medium that contains 20% conditioned medium from MSCs (20% MSC-CM) or from CAFs (20% CAF-CM) had similar strong promoting effects on the growth of individual colonies in the presence of fulvestrant (Figure 1E). Unlike MSCs, 20% MSC-CM or 20% CAF-CM had also some moderate effects on colony growth in the absence of fulvestrant. In a different set of experiments with an ATP-based growth assay, cell growth was monitored after cells had been seeded at higher cell density that prevented the formation of individual colonies, but still left sufficient space for cell growth over a growth period of five days. Also under these conditions, CAF-CM was able to strongly increase cell growth in the presence of fulvestrant, but failed to promote growth or even reduced growth in the absence of fulvestrant (Figure 1F).

We next sought to analyze whether stromal cells also affect spheroid formation. We found that 20% CAF-CM increases the size of spheroids both in the presence and absence of fulvestrant (Figure 1G). However, these effects did not seem to be the result of increased MCF-7 cell growth. It rather seemed that CAF-CM causes the MCF-7 cells in the spheroids to be more loosely packed (Figure 1G) This may be the result of a reduced cell-cell contact as was found in spheroids that MCF-7 cells had formed in the presence of MSCs [35]. Next, we examined whether CAF-CM affected the expression of mesenchymal and stem cell markers. Of the selected markers, only fibronectin-1 showed increased RNA levels upon treatment with CAF-CM both in the presence and absence of fulvestrant (Figure 1H). In the absence of fulvestrant, also the expression of ALDH3A1 was increased by CAF-CM.

Collectively, these data suggest that MSCs and CAFs secrete certain factors that protect MCF-7 cells from the growth-inhibitory effect of fulvestrant. Since most of the selected mesenchymal and stem cell markers were not affected by CAF-CM, it is unlikely that the stroma cell-induced protective effect against fulvestrant is mediated by an increase in the stem/progenitor cell pool of the MCF-7 cells.

### MSC- and CAF-CM interfere with the activities of signaling pathways and the expression of proteins involved in drug resistance

To identify the mechanism that underlies the MSC/CAF-induced fulvestrant resistance, we examined the expression of a number of proteins, integrin β1, IGF1R and Bcl-3, and the activities of a number of signaling pathways, the PI3K/AKT-, the Ras/Raf/MEK1/ERK1/2-pathways, the JAK2 (janus kinase 2)/STAT3 (signal transducer and activator of transcription 3) and the hypoxia-regulated pathway. All these proteins and signaling pathways have been associated with drug resistance and/or deregulation of ERα activity [14, 16, 17, 21, 28, 36, 37]. To study the activities of the four pathways we determined the phospho-protein levels of AKT, ERK1/2, STAT3 and the plasma-membrane level of the hypoxia-regulated protein CAIX (carbon anhydrase) [38] by Western blot analysis. To mimic potential effects on the PI3K/AKT- and the Ras/Raf/MEK1/ERK1/2- pathways by stromal cells we used insulin, shown to induce these pathways in MCF-7 cells [39]. To recapitulate potential stromal cell effects on the hypoxia-regulated pathway we used the hypoxia-mimetic agent CoCl_2_.

**Figure 2:**
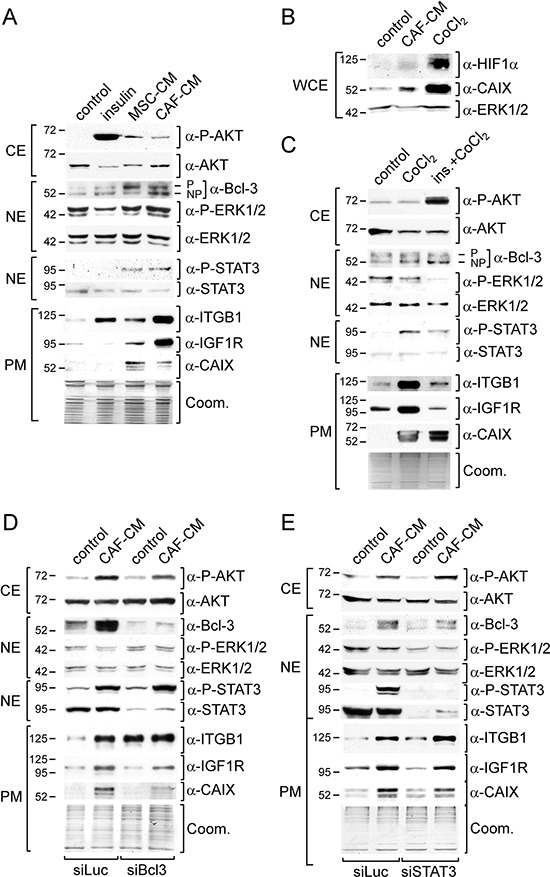
MSC- and CAF-CM upregulate the activities of signaling pathways and expression of proteins relevant in the acquisition of drug resistance Western blot analyses of the indicated proteins and phospho-proteins were performed after MCF-7 cells were incubated with insulin, 100 μM CoCl_2,_ insulin plus CoCl_2_ (ins./CoCl_2_), MSC- or CAF-CM and/or transfected with siBcl3, siSTAT3 or with the control siRNA siLuc. Depending on the cellular location of the protein or phospho-protein, either the cytosolic (CE), nuclear (NE) or plasma membrane (PM) extract was used for the analysis **A–C, E.** For the visualization of the HIF1α protein, whole cell extracts (WCE) were prepared by lysing cells in RIPA buffer **B.** To check for equal loading of plasma membrane proteins, proteins remaining in the gel after blotting were stained with Coomassie Blue (Coom.).

We found that a 3-day-incubation of MCF-7 cells with 20% MSC- or 20% CAF-CM similarly increased phosphorylation of AKT and STAT3, while having no effect on ERK1/2 phosphorylation (Figure 2A). Both CMs also increased plasma membrane abundance of CAIX. As shown with CAF-CM, also the level of CAIX-regulator HIF1α (hypoxia inducible factor 1α) was raised (Figure 2B). This suggests that MSC- and CAF-CM activate the PI3K/AKT-, the JAK2/STAT3- and the hypoxia-regulated pathway, whereas it had no effect on the Ras/Raf/MEK/ERK1/2 pathway. However, limiting the duration of incubation with CAF-CM to overnight led to an increase in the P-ERK1/2 levels (Supplementary Figure S1) suggesting that the activation of the Ras/Raf/MEK/ERK1/2 pathway by stromal cell CM is temporary. MSC- and CAF-CM also increased the plasma membrane levels of IGF1R and integrin β1 and the nuclear protein level of Bcl-3 (Figure 2A). Of note, the two Bcl-3-specific protein bands likely correspond to a phosphorylated and the non-phosphorylated form of Bcl-3, of which both are able to regulate transcription [40]. Insulin could mimic the effect of MSC- and CAF-CM on P-AKT and integrin β1 (Figure 2A). Also, like stromal cell-CM, insulin induced a temporary increase in ERK1/2 phosphorylation (Supplementary Figure S1). However, unlike stromal cell-CM, insulin did not modulate the levels of P-STAT3, Bcl-3, IGF1R and CAIX (Figure 2A). The hypoxia-mimetic agent CoCl_2_, that strongly induced the expression of HIF1α protein und CAIX in MCF-7 cells (Figure 2B), shared with insulin the ability to increase the integrin β1 level and also, like insulin, had no effect on Bcl-3 expression (Figure 2C). However, unlike insulin, CoCl_2_ recapitulated the effect of MSC- and CAF-CM on CAIX, P-STAT3, IGF1R expression, while having no effect on P-AKT (Figure 2C). Consequently, when combined, insulin and CoCl_2_ could mimic most of the effects of MSC- and CAF-CM. The only two tested stroma cell-responsive proteins whose expression remained unchanged in response to a combined treatment with insulin and CoCl_2_ were Bcl-3 and IGF1R.

To test whether Bcl-3 and IGF1R expression are linked, we downregulated Bcl-3 by a Bcl-3-specific siRNA (siBcl3). In fact, siBcl3 attenuated the effect of CAF-CM on the expression of IGF1R and also on that of CAIX, while it strongly increased the integrin β1 level (Figure 2D). This suggests that stromal cells induce the expression of IGF1R and also CAIX by upregulating the Bcl-3 level. Since STAT3 can regulate Bcl-3 expression in prostate cancer [41], we wondered whether STAT3 and Bcl-3 expression are also linked in MCF-7 cells. However, knock-down of STAT3 by a STAT3-specific siRNA (siSTAT3) only slightly reduced CAF-CM-induced Bcl-3 expression and, with it, also weakly diminished the levels of IGF1R and CAIX (Figure 2E). Hence, activation of STAT3 by stromal cell CM is unlikely to be the major cause for CM-induced Bcl-3 expression, although it may contribute to it.

### MSCs and CAFs coordinately modulate signaling pathways and protein expression by downregulating the IGFBP5 level

**Figure 3:**
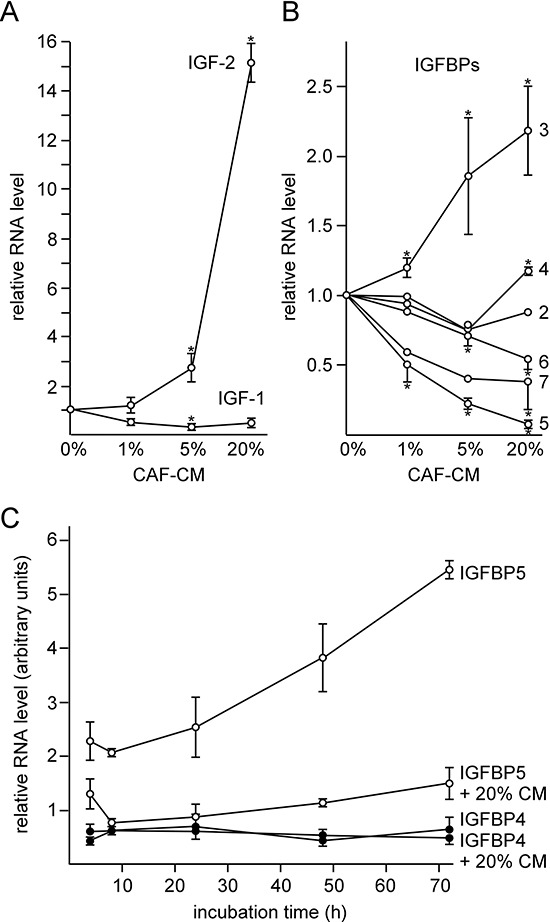
CAF-CM induces the downregulation of IGFBP5 expression **A, B.** RT-PCR analyses of RNA isolated from MCF-7 cells after 2-day-exposure to 0, 1, 5 or 20% CAF-CM for the abundance of IGF-1/-2 specific mRNAs (A) and IGFBP1-7-specific mRNAs (B). **C.** Time-course study to measure changes in IGFBP4/5 mRNA levels in MCF-7 cells within 4–72 h after addition of 20% CAF-CM or no addition of CAF-CM.

Next we analyzed the involvement of the IGF1R-dependent signal pathway in the MSC- and CAF-induced effects for two reasons. One, MSC- and CAF-CM upregulated the IGF1R level along with the P-AKT level. Two, blockage of IGF1R-dependent signaling by the IGF1R-specific inhibitor PQ401 strongly reduced the phosphorylation status of AKT (Supplementary Figure S1). suggesting that IGF1R is a major driver of the PI3K/AKT pathway in MCF-7 cells. IGF1R activity is regulated by its activators IGF1 and 2 and by the IGF-binding proteins IGFBP1-6 which regulate the activity of the IGFs. Besides their IGF-dependent activities, IGFBPs also show IGF-independent effects [19]. Therefore, in theory, by modulating IGFBP expression, stromal cells could modulate IGF-dependent and -independent effects at the same time. Hence, we analyzed whether CAF-CM is able to induce changes in the expression of any of the IGFs and the IGFBPs by quantitative RT-PCR. We also included IGFBP7 in our analysis, since IGFBP7 was shown to bind to IGF1R and to block its activation by IGFs [42]. When we incubated MCF-7 cells with increasing amounts of CAF-CM (1, 5 and 20%), the mRNA level of IGF2, which showed higher basal levels than IGF1 (data not shown), increased up to ~15-fold (Figure 3A). Of the IGFBPs, IGFBP5, 6 and 7 showed a dose-dependent decline in their expression in response to CAF-CM, whereas the level of IGFBP3 increased in the presence of CAF-CM (Figure 3B). Of all IGFBPs, IGFBP5 was expressed at the highest basal level in MCF-7 cells, followed by IGFBP4 and IGFBP2 (data not shown). The other four IGFBPs were much less abundant with IGFBP1 showing the lowest (barely detectable) expression. Therefore, of all CAF-CM-induced changes in IGFBP expression, the changes in the IGFBP5 levels should be most relevant for IGF1R activity. Hence, we focused our further analysis on IGFBP5. We found that the change in the IGFBP5 level by CAF-CM was an early event. Four hours of incubation of MCF-7 cells with CAF-CM was sufficient to bring IGFBP5 expression down to ~50% of its original level (Figure 3C). In addition, the suppressive effect of CAF-CM on IGFBP5 levels was long-lasting.

**Figure 4:**
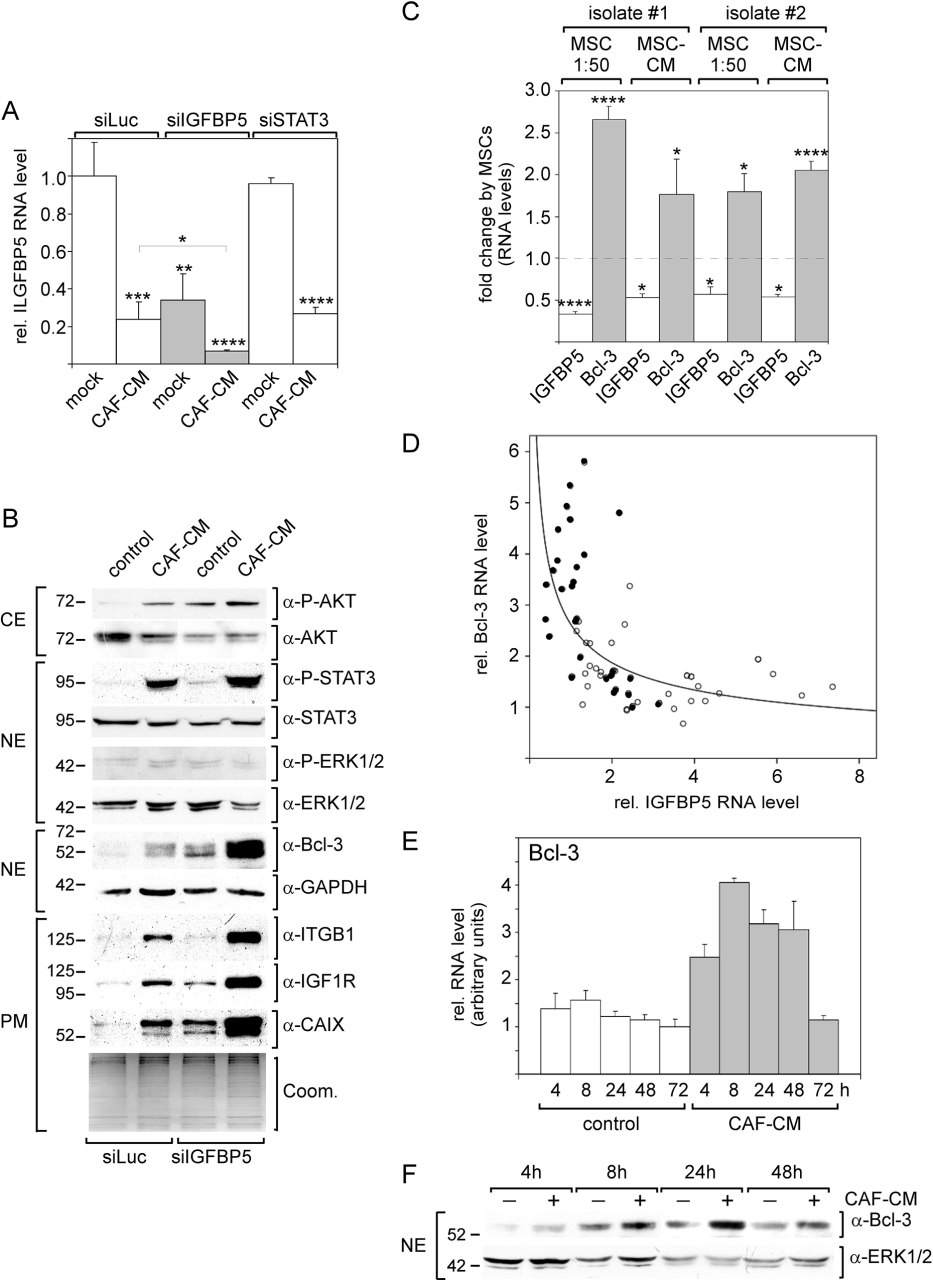
Knock-down of IGFBP5 mimics most of the CAF-CM-induced effects on signaling pathways and protein expression **A.** RT-PCR analysis of IGFBP5 mRNA levels in MCF-7 cells transfected with siIGFBP5, siSTAT3 or siLuc (control siRNA) followed by treatment with CAF-CM or no treatment. **B.** Western blot analyses of levels of stromal cell-regulated proteins and phospho-proteins after treatment of MCF-7 cells with siIGFBP5 or siLuc in the presence or absence of CAF-CM (CE = cytosolic extract, NE = nuclear extract, PM = plasma membrane extract). To check for equal loading of plasma membrane proteins, proteins remaining in the gel after blotting were stained with Coomassie Blue (Coom.) **C.** Effects of two different human MSCs isolates on IGFBP5 and Bcl-3 levels in MCF-7 cells. Either MSCs were co-cultured with MCF-7 cells in a ratio of 1:50 or 20% MSC-CM was added to the MCF-7 cells. **D.** RT-PCR analyses of RNAs isolated from MCF-7 cells treated with CAF-CM (•) or from untreated MCF-7 cells (○) for IGFBP5 and Bcl-3 mRNA levels. **E, F.** Comparison of the Bcl-3 mRNA RNA (E) and protein (F) levels in the presence and absence of CAF-CM in a time course experiment. For each time point, the difference in Bcl-3 expression between control cells and CAF-CM-treated cells is statistically significant as determined by paired sample student's *t*-test. In (A, D, F), each bar represents the mean value ± S.D. of at least three independent experiments.

To analyze whether downregulation of IGFBP5 is sufficient to induce the observed effects of MSCs and CAFs on MCF-7 cells, MCF-7 cells were treated with an IGFBP5-specific siRNA (siIGFBP5). siIGFBP5 decreased IGFBP5 levels by ~4-fold, which was comparable to the decline in IGFBP5 expression as observed in response to CAF-CM (Figure 4A). Of note, siSTAT3 had no effect on IGFBP5 expression suggesting that stromal cell-induced activation of the JAK2/STAT3 pathway is not responsible for the downregulation of the IGFBP5 level. A comparison of the effects of siIGFBP5 and CAF-CM revealed that siIGFBP5 was as effective as CAF-CM to upregulate the levels of P-AKT, Bcl-3 and CAIX (Figure 4B). In addition, siIGFBP5 slightly increased the levels of IGF1R and P-STAT3, whereas siIGFBP5 alone failed to modulate the expression of integrin β1. Interestingly, treatment of MCF-7 cells with both CAF-CM and siIGFBP5, which reduced IGFBP5 expression to ~7% of its original level (Figure 4A), generated the highest levels of Bcl-3, IGF1R, CAIX, integrin β1 and P-STAT3 (Figure 4B). These data suggest that stromal cells can induce most of the observed effects on signaling pathway activity and protein expression by simply downregulating IGFBP5 expression.

### Bcl-3 is involved in stromal cell-induced fulvestrant resistance

The strong effect of siIGFBP5 on the Bcl-3 level prompted us to further analyze the link between IGFBP5 and Bcl-3 expression. First, we wanted to confirm that MSCs also downregulate the IGFBP5 level while upregulating that of Bcl-3. For this, we used two MSC isolates and either co-cultured MSCs with MCF-7 cells in a ratio of 1:50 or added 20% MSC-CM to the MCF-7 cells. Under all conditions, both MSC isolates significantly downregulated IGFBP5 RNA expression and, at the same time, upregulated the Bcl-3 RNA level (Figure 4C). We next compared IGFBP5 levels with those of Bcl-3 in 76 RNA samples isolated from MCF-7 cells either treated with CAF-CM (closed circles, N = 32) or left untreated (open circles, N = 44). The data suggest an exponential, inverse correlation between the expression of both genes such that changes in IGFBP5 expression have the most profound effects on Bcl-3 expression when IGFBP5 levels are low (Figure 4D). The hypothesis that IGFBP5 and Bcl-3 expression are linked is further supported by the observation that CAF-CM modulated Bcl-3 mRNA and protein expression early (Figure 4E, 4F), just as seen with IGFBP5 (Figure 3C).

**Figure 5:**
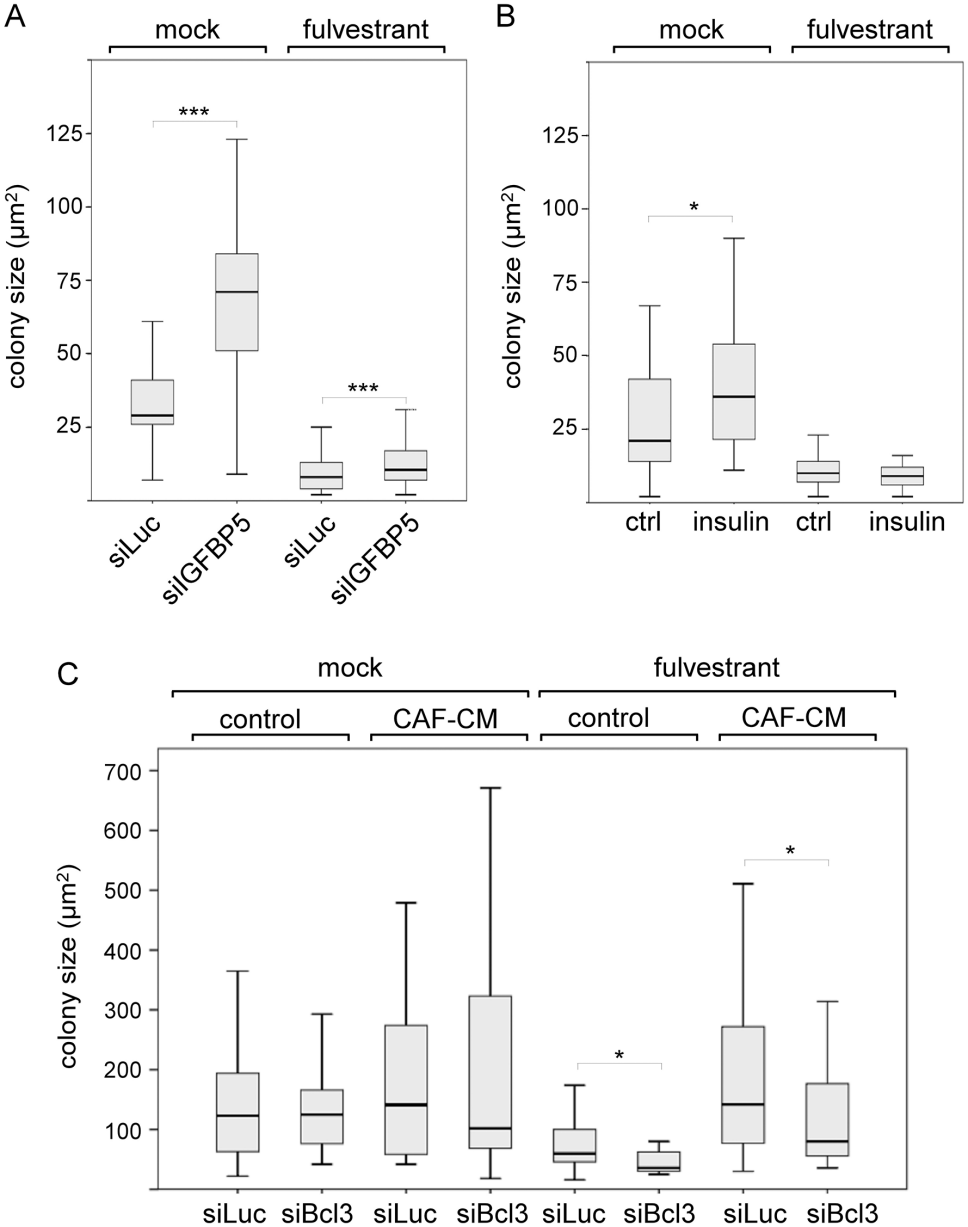
Bcl-3 knock-down specifically interferes with MCF-7 growth in the presence of fulvestrant **A, C.** Effect of siIGFBP5 (A), siBcl3 (C) and siLuc (A, C) on the size of individual colonies of MCF-7 cells in the clonogenic assay in the presence or absence of fulvestrant and in the presence or absence of CAF-CM. **B.** Clonogenic assays performed with insulin- or mock-treated MCF-7 cells. Statistical analyses were carried out by using the Wilcoxon test.

Having established that downregulation of IGFBP5 allows MSCs and CAFs to coordinate a number of events, we explored the possibility that a decline in the IGFBP5 level also affects colony growth of MCF-7 cells. By using siIGFBP5, we found that a decrease in IGFBP5 expression resulted in an increase in average colony size both in the absence and presence of fulvestrant (Figure 5A). To check whether the PI3K/AKT pathway was responsible for these effects, we repeated the experiments with insulin. While insulin was able to significantly increase average colony growth in the absence of fulvestrant, it failed to do so in the presence of fulvestrant (Figure 5B). This suggests that the effect of siIGFBP5 on growth in the presence of fulvestrant was not caused by the activation of the PI3K/AKT pathway. Next, we tested whether Bcl-3 may affect MCF-7 cell growth. Strikingly, siBcl-3 did not significantly alter cell growth in the absence of fulvestrant, but selectively reduced growth in the presence of fulvestrant, most profoundly in the presence of CAF-CM (Figure 5C). This suggests that the increased expression of Bcl-3 caused by stromal cell-induced downregulation of IGFBP5 is at least partially responsible for stromal cell-mediated fulvestrant resistance.

### Downregulation of IGFBP5 has an impact on the expression of stromal cell-regulated genes

We next sought to identify stromal cell-regulated MCF-7 cell genes that are inversely regulated by IGFBP5 and Bcl-3. For this reason, we performed two sets of independent cRNA microarray experiments comparing the transcripts of MCF-7/MSC co-cultures (ratio: 50 to 1) with those in MCF-7 cells alone. The expression of a total of 253 mRNA species was changed by ≥2-fold in the presence of MSCs (Supplementary Table S2). Of these, 18 mRNA species showed reduced expression in the MCF-7/MSC co-cultures. The much higher number of upregulated RNA species could at least partly be explained by the presence of MSC-derived RNAs, such as PAI-1 (plasminogen activator inhibitor-1)-specific, ZEB-1 (zinc finger E-box binding homeobox 1)-specific or CD90-specific RNAs, that are strongly expressed in MSCs, but weakly or not expressed in MCF-7 cells (data not shown). For further analysis, we focussed on the RNAs that showed lower expression in the MCF-7/MSC co-cultures, since, in theory, they should derive from MCF-7 cells. Of the 18 downregulated mRNA species, 17 coded for known genes. For 13 of these 17 genes MSC-dependent changes could be confirmed by Q-RT-PCR assays. Of these 13 genes, two genes (CDSN and CALCR) showed very low expression and were therefore excluded from further analysis. The remaining 11 genes are listed in Table 1. Nine of these genes were also downregulated in MCF-7/MSC co-cultures when cells were kept in 3D suspension cultures that led to spheroid formation. Ten of these genes showed also reduced expression in MCF-7/MSC co-cultures when MSCs and MCF-7 cells were separated by a 0.4 μm filter that prevented direct cell-cell contact, but allowed paracrine effects. MSC-CM induced the level of nine of these genes, while CAF-CM reduced the expression of all eleven genes. Interestingly, most of these genes were also responsive to insulin and CoCl_2_.

**Table 1:** Relative expression of selected MSC/CAF-responsive genes in MCF-7 cells under different conditions

gene	relative RNA expression*									
	MCF7/MSC co-culture 50:1, (#)	MCF7/MSC co-culture 10:1, 3D	MCF7/MSC TW (0.4 μm) 10:1	MSC-CM 20%	CAF-CM 20%	siIGFBP5 vs. siLuc	siBcl3 vs. siLuc	siSTAT3 vs. siLuc	Insulin vs. mock	CoCl_2_ vs. mock
**KLHL4**	**0.35 ± 0.19** (*p* = 0.018)	**0.06 ± 0.01** (*p* = 0.02)	**0.14 ± 0.03** (*p* = 0.0026)	**0.28 ± 0.05** (*p* = 0.0055)	**0.26 ± 0.08** (*p* = 0.0033)	**0.49 ± 0.26** (*p* = 0.036)	**2.46 ± 0.68** (*p* = 0.022)	**0.72 ± 0.09** (*p* = 0.0052)	**0.50 ± 0.04** (*p* = 0.011)	**0.59 ± 0.11** (*p* = 0.030)
**SEPP1**	**0.39 ± 0.20** (*p* = 0.013)	**0.14 ± 0.02** (*p* = 0.0034)	**0.54 ± 0.17** (*p* = 0.050)	0.86 ± 0.05 (*p* = 0.3)	**0.25 ± 0.01** (*p* < 0.0001)	**0.55 ± 0.11** (*p* = 0.03)	**2.66 ± 0.32** (*p* = 0.0018)	**1.53 ± 0.16** (*p* = 0.027)	**0.47 ± 0.19** (*p* = 0.0087)	**0.31 ± 0.06** (*p* = 0.0015)
**TMEM26**	**0.39 ± 0.24** (*p* = 0.0038)	**0.53 ± 0.10** (*p* = 0.037)	**0.24 ± 0.16** (*p* = 0.008)	**0.38 ± 0.15** (*p* = 0.0026)	**0.47 ± 0.06** (*p* = 0.0028)	**0.56 ± 0.15** (*p* = 0.0086)	1.22 ± 0.07 (*p* = 0.16)	**0.75 ± 0.05** (*p* = 0.0054)	0.83 ± 0.19 (*p* = 0.42)	**0.48 ± 0.16** (*p* = 0.013)
**TGFBR3**	**0.53 ± 0.19** (*p* = 0.024)	**0.36 ± 0.08** (*p* = 0.044)	**0.30 ± 0.06** (*p* = 0.0008)	**0.35 ± 0.07** (*p* = 0.0012)	**0.45 ± 0.04** (*p* = 0.011)	0.64 ± 0.35 (*p* = 0.19)	0.98 ± 0.08 (*p* = 0.83)	1.00 ± 0.17 (*p* = 1.00)	**0.61 ± 0.04** (*p* = 0.034)	**0.54 ± 0.08** (*p* = 0.013)
**RAB30**	**0.52 ± 0.22** (*p* = 0.00012)	**0.36 ± 0.03** (*p* = 0.008)	**0.37 ± 0.06** (*p* = 0.0009)	0.74 ± 0.09 (*p* = 0.16)	**0.28 ± 0.05** (*p* = 0.0024)	0.65 ± 0.28 (*p* = 0.12)	1.67 ± 0.37 (*p* = 0.055)	0.87 ± 0.16 (*p* = 0.52)	**0.25 ± 0.04** (*p* = 0.002)	**0.81 ± 0.05** (*p* = 0.013)
**FGF18**	**0.45 ± 0.25** (*p* = 0.012)	**0.33 ± 0.02** (*p* < 0.0001)	**0.21 ± 0.06** (*p* = 0.0002)	**0.40 ± 0.09** (*p* = 0.037)	**0.35 ± 0.06** (*p* = 0.0054)	0.66 ± 0.54 (*p* = 0.35)	1.16 ± 0.09 (*p* = 0.20)	1.16 ± 0.47 (*p* = 0.47)	0.67 ± 0.14 (*p* = 0.075)	**0.55 ± 0.06** (*p* = 0.0008)
**KLK11**	**0.34 ± 0.10** (*p* = 0.0018)	**0.07 ± 0.02** (*p* = 0.0071)	**0.19 ± 0.12** (*p* = 0.0005)	**0.22 ± 0.04** (*p* = 0.035)	**0.16 ± 0.01** (*p* = 0.0012)	0.87 ± 0.20 (*p* = 0.20)	1.22 ± 0.23 (*p* = 0.22)	**1.24 ± 0.09** (*p* = 0.015)	**0.53 ± 0.21** (*p* = 0.041)	**0.45 ± 0.04** (*p* = 0.0018)
**UGT2B15**	**0.36 ± 0.19** (*p* = 0.0059)	**0.13 ± 0.06** (*p* = 0.0067)	**0.16 ± 0.03** (*p* = 0.0026)	**0.34 ± 0.09** (*p* = 0.0013)	**0.16 ± 0.01** (*p* = 0.00091)	0.89 ± 0.15 (*p* = 0.38)	**3.60 ± 0.28** (*p* = 0.0004)	1.41 ± 0.38 (*p* = 0.17)	**0.44 ± 0.19** (*p* = 0.020)	**0.64 ± 0.16** (*p* = 0.025)
**KIF12**	**0.60 ± 0.17** (*p* = 0.01)	**0.24 ± 0.09** (*p* = 0.0016)	**0.34 ± 0.13** (*p* = 0.011)	**0.30 ± 0.01** (*p* < 0.0001)	**0.31 ± 0.03** (*p* = 0.011)	0.99 ± 0.51 (*p* = 0.96)	**0.55 ± 0.09** (*p* = 0.0034)	1.24 ± 0.18 (*p* = 0.15)	**0.37 ± 0.06** (*p* = 0.015)	**0.42 ± 0.14** (*p* = 0.019)
**RAMP3**	**0.51 ± 0.41** (*p* = 0.021)	0.69 ± 0.40 (*p* = 0.27)	**0.41 ± 0.15** (*p* = 0.001)	**0.33 ± 0.09** (*p* = 0.023)	**0.30 ± 0.04** (*p* = 0.0086)	**0.32 ± 0.24** (*p* = 0.013)	**0.59 ± 0.10** (*p* = 0.022)	**0.64 ± 0.09** (*p* = 0.023)	**0.39 ± 0.06** (*p* = 0.015)	**0.37 ± 0.07** (*p* = 0.012)
**YPEL-1**	**0.44 ± 0.26** (*p* = 0.018)	0.83 ± 0.20 (*p* = 0.4)	0.81 ± 0.04 (*p* = 0.17)	**0.55 ± 0.08** (*p* = 0.06)	**0.35 ± 0.09** (*p* = 0.028)	**0.23 ± 0.19** (*p* = 0.010)	0.88 ± 0.32 (*p* = 0.61)	0.62 ± 0.19 (*p* = 0.25)	**0.21 ± 0.10** (*p* = 0.015)	0.63 ± 0.13 (*p* = 0.082)

* expression relative to control condition as measured by Q-RT-PCR after 2 days of incubation. Statistically significant changes are marked in bold. Genes are ordered by the strength of their response to siIGFBP5. RAMP3 and YPEL-1 are listed separately, as their expression is not significantly changed in response to MSCs in 3D spheroid cultures.

(#) condition as used for cRNA microarray analysis. TW = transwell.

**KLHL4** = kelch-like 4, **SEPP1** = selenoprotein P. plasma 1, **TMEM26** = transmembrane protein 26, **TGFBR3** = transforming growth factor β receptor III, **RAB30** = RAB30, member RAS oncogene family, **FGF18** = fibroblast growth factor 1, **KLK11** = kallikrein-related peptidase 1, **UGT2B15** = UDP glucuronosyl transferase 2 family polypeptide B15, **KIF12** = kinesin family member 12, **RAMP3** = receptor (G protein-coupled) activity modifying protein 3, **YPEL-1** = yippee-like 1

Collectively, these data suggest that these eleven genes are regulated by MSCs and CAFs through soluble factors that these stromal cells secret, just as was found for the stroma cell-mediated regulation of IGFBP5 and Bcl-3.

**Figure 6:**
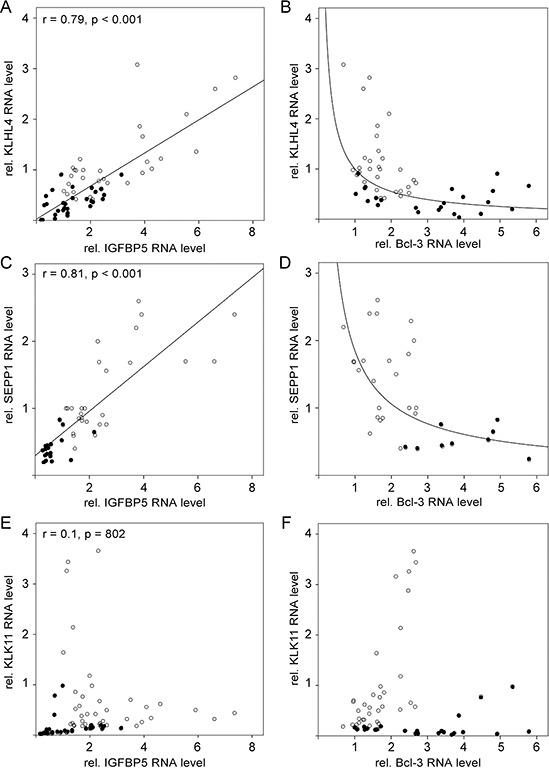
KLHL4 and SEPP1 mRNA levels change along with the IGFBP5 mRNA level in a linear fashion **A–F.** RT-PCR analyses of RNA isolated from MCF-7 cells either exposed to CAF-CM (•) or left untreated (○). Levels of KLHL4-, SEPP1- and KLK11-specific mRNAs were either compared with the mRNA level of IGFBP5 (A, C, E) or with the mRNA level of Bcl-3 (B, D, F).

Examining the expression of these genes in the presence of siIGFBP5 and siBcl3, we found that two genes (SEPP1 and KLHL4) were inversely regulated by siIGFBP5 and siBcl3. Like stromal cells, siIGFBP5 significantly downregulated the expression of these two genes, while siBcl3 upregulated their levels. Moreover, the expression of KLHL4 and SEPP1 correlated well with that of IGFBP5 (Figure 6A, 6C) and showed an inverse correlation to that of Bcl-3 (Figure 6B, 6D). As a control we used KLK11. This stromal cell-regulated gene was neither affected by siIGFBP5 nor by siBcl-3 (Table 1). Nor did the expression of this gene show any correlation with the expression of IGFBP5 or Bcl-3 (Figure 6E, 6F).

These data suggest that two MSC/CAF-regulated genes, KLHL4 and SEPP1, are inversely regulated by IGFBP5 and Bcl-3.

### Stromal cell-induced downregulation of IGFBP5 in other breast cancer cells

**Figure 7:**
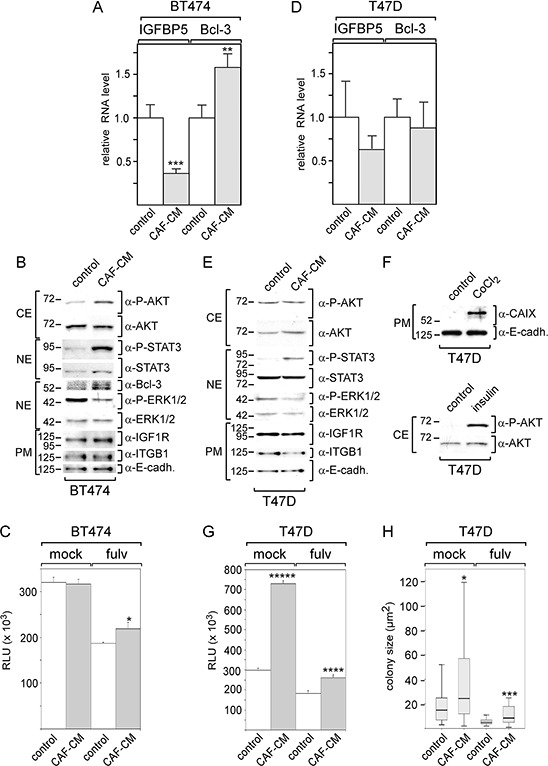
BT474, but not T47D cells respond to CAF-CM by changing IGFBP5 and Bcl-3 levels and by increasing growth activity in the presence of fulvestrant **A–D.** After BT474 cells (A, B) or T47D cells (D, **E.**) were treated with CAF-CM or left untreated for three days, RNA or proteins were isolated and analyzed for IGFBP5 and Bcl-3 RNA levels (A, D) or levels of certain proteins as indicated (B, E), respectively. To check for equal loading of plasma membrane proteins, blots were reprobed with an E-cadherin -specific antibody (α-E-cadh.). **F.** T47D cells were also tested for AKT phosphorylation and CAIX expression after 3-day-treatment with insulin or CoCl_2_, respectively. (C, **G, H.**) Effect of CAF-CM on BT474 and T47D cell growth in the presence or absence of fulvestrant as measured either by an ATP based assay (C, G) or by measuring the sizes of single colonies (H). In (A, C, D, G), each bar represents the mean value ± S.D. of at least three independent experiments, in (H), the data of a representative experiment are shown. Statistical analysis was either performed by using the student's *t*-test (A, C, D, G) or the Wilcoxon test (H).

We next examined whether stromal cells may be able to modulate signal pathway activities and protein expression also in other ERα-positive breast cancer cell lines (BT474 and T47D) in a similar way as in MCF-7 cells and whether they may also affect cell growth in the presence of fulvestrant. As seen with MCF-7 cells, BT474 cells responded to CAF-CM by a significant decrease in the IGFBP5 RNA expression and a significant rise in the Bcl-3 RNA level (Figure 7A). CAF-CM also increased Bcl-3 protein expression and upregulated the levels of the P-AKT and P-STAT3 (Figure 7B). However, in contrast to MCF-7 cells, BT474 cells failed to increase the expression of integrin β1 and IGF1R in response to CAF-CM. Of note, insulin also failed to increase the integrin β1 level in BT474 cells (data not shown). By being much higher in BT474 cells than in MCF-7 cells, the level of integrin β1 may have already reached its maximum value in its basal state. It is also noteworthy that the CAIX protein could not be detected by Western blot analysis, even when cells were treated with CoCl_2_ (data not shown). In an ATP-based growth assay, CAF-CM significantly promoted BT474 growth in the presence of fulvestrant, though the effect was less pronounced than that seen with MCF-7 cells. Importantly, no effect of CAF-CM could be observed on BT474 cell growth in the absence of this drug (Figure 7C). Of note, clonogenic assay could not be performed with BT474 cells, since BT474 cells did not survive when seeded at low density.

Collectively, these data indicate that BT474 cells behave similar to MCF-7 cells in terms of their responses to CAF-CM. Like MCF-7 cells, BT474 cells show higher growth activity in the presence of fulvestrant, lower expression of IGFBP5, higher levels of Bcl-3 and an increase in the PI3K/AKT pathway activity suggesting that CAF-CM induces these changes in both cell lines through the same mechanism.

In contrast to BT474 and MCF-7 cells, T47D cells were quite unresponsive to CAF-CM in terms of changes in protein expression and pathway activities (Figure 7D, 7E). CAF-CM only increased the P-STAT3 levels in T47D cells (Figure 7E). Of note, Bcl-3 protein could not be detected (data not shown). To check whether the PI3K/AKT and HIF1α/CAIX pathways are functional in T47D cells, we treated these cells with insulin and CoCl_2_. Insulin and CoCl_2_ were able to raise the P-AKT level or CAIX level, respectively, (Figure 7F) demonstrating that the failure of CAF-CM to induce insulin- and CoCl_2_-like effects in T47D cells was not due to unresponsiveness of the PI3K/AKT and HIF1α/CAIX pathways. In ATP- and colony growth assays, CAF-CM showed a promoting effect on T47D cell growth irrespective of whether fulvestrant was present or not (Figure 7G, 7H).

The data obtained with T47D cells suggest that the responses of ERα-positive breast cancer cells to stromal cells can differ.

### The expression of Bcl-3 is associated with an unfavorable outcome of endocrinally treated breast cancer patients with ERα/PR-positive tumors

**Figure 8:**
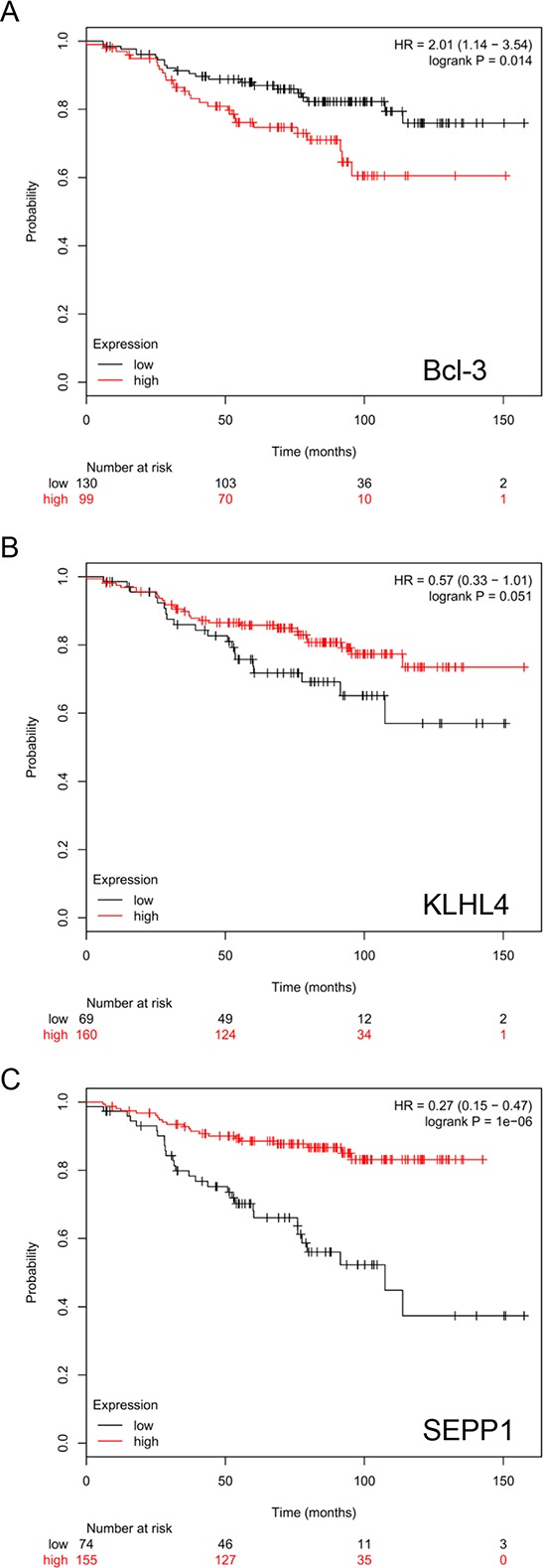
Higher Bcl-3 expression is associated with an unfavorable prognosis of endocrinally treated breast cancer patients with ERα+/PR+-tumors *In silico*-Kaplan-Meier-Plotter analysis of 229 endocrinally treated breast cancer patients with ERα^+^/PR^+^-tumors for an association of outcome (relapse-free survival) with Bcl-3 **A.** KLHL4 **B.** or SEPP1 **C.** expression by choosing best cut-off.

We next sought to analyze whether Bcl-3 is associated with the outcome of breast cancer patients that suffered from ERα/PR-positive tumors and received endocrine treatment. For this reason, we performed an *in-silico* analysis by using the Kaplan-Meier-Plotter available under http://kmplot.com/analysis/index.php?p=service&default=true which is based on data published by Gyorffy et al. [43]. The selected criteria (ERα^+^/PR^+^/received endocrine treatment) were met by a cohort of 229 patients. The analysis for this cohort revealed that higher Bcl-3 mRNA levels were significantly associated with a more unfavorable relapse-free survival (Figure 8A). We also run the analysis for KLHL4 and SEPP1, whose expression could be increased by siBcl-3 and showed an inverse correlation to that of Bcl-3. For these two genes, higher mRNA levels correlated with a better relapse-free survival (Figure 8B, 8C). While the data for KLHL4 slightly missed the significance level (*p* = 0.051) (Figure 8B), those for SEPP1 were highly significant (Figure 8C). Collectively, these data are consistent with the notion that Bcl-3 is involved in endocrine resistance and that KLHL4 and SEPP1 are negatively regulated by Bcl-3. The data might also suggest that Bcl-3 mediates endocrine resistance at least partially by downregulating KLHL4 and SEPP1.

## DISCUSSION

The data presented here show that MSCs and CAFs affect ERα-positive breast cancer cells in a similar way. They promote cellular growth in the presence of the anti-estrogen fulvestrant and change the activities of the same signaling pathways and the expression of the same proteins. Most of the changes in signaling pathway activities and protein expression could be recapitulated by combined treatment with insulin and CoCl_2_. Only stromal cell-induced changes in IGF1R and Bcl-3 expression could not be mimicked by a combination of these agents. We present evidence that stromal cells induce all of these changes by downregulating IGFBP5 expression. Downregulation of IGFBP5 also contributed to the activation of STAT3. It seems therefore that, by downregulating IGFBP5, MSCs and CAFs coordinately induce a wide range of changes in breast cancer cells.

Since some changes as induced by IGFBP5 downregulation could be mimicked by insulin, some by CoCl_2_ and others neither by insulin nor CoCl_2_, it is likely that IGFBP5 fulfills several different functions in breast cancer cells. Besides its classical function as a regulator of IGF-dependent IGF1R activation [44], IGF1R-independent actions of IGFBP5 have been described [19]. E.g., it has been shown that, by binding to integrin α2/β1, IGFBP5 is able to regulate cell adhesion, migration and survival of MCF-7 cells [20]. This activity did not require the N-terminal domain, which is responsible for its IGF1R-depending action, but the C-terminal domain, which interacts with heparin. The notion that the N- and C-terminal domains of IGFBP5 have different functions is also supported by a study that showed that the N-terminal domain blocks proliferation of osteosarcoma cells and induces apoptosis, whereas the C-terminal domain inhibits migration and invasion [45]. In addition, the C-terminal domain contains a nuclear localization sequence allowing IGFBP5 to fulfill functions in the nucleus [46]. IGF1R-independent functions of IGFBP5 have also been found *in-vivo* studies [47] just further substantiating the hypothesis that at least two independent activities of IGFBP5 exist.

Downregulation of IGFBP5 by siIGFBP5 resulted in increased cellular growth both in the presence and absence of fulvestrant. The effect of siIGFBP5 on cell growth in the absence of fulvestrant could be mediated by its insulin-like effect on P-AKT, since insulin was able to foster cell growth in absence, but not in the presence of fulvestrant. The effect of siIGFBP5 on cell growth in the presence of fulvestrant is likely mediated by upregulation of Bcl-3 expression, since siBcl-3 specifically attenuated cellular growth in the presence of fulvestrant, while showing no effect on growth in its absence. Hence, Bcl-3 may protect ERα-positive breast cancer cells, such as MCF-7 cells, against fulvestrant. In line with this hypothesis, a previous study on MCF-7 cells grown in ovariectomized mice showed that estrogen deficiency was linked to higher Bcl-3 expression [28]. In this study, along with the higher expression of Bcl-3, a higher DNA-binding activity of NFκB was observed. By binding to p50/p50 and p52/p52 NFκB homodimers, Bcl-3 is thought to increase NFκB activity [27]. Thus, by upregulating Bcl-3 levels in ERα-positive breast cancer cells, MSCs and CAFs may pave the way towards an NFκB-dependent growth under conditions where ERα is not functional. Interestingly, NFκB activation does not only protect against loss of ERα function, but also promotes growth of Her2-positive breast cancer cells in the presence of a Her2 inhibitor [48] suggesting that NFκB activation is a general option for breast cancer cells to survive under conditions where the major growth-permitting pathway is blocked. In line with this notion, ERα-negative breast cancer cells are found to be more dependent on NFκB activity for proliferation than ERα-positive cells [49].

We could show that stromal cell-induced downregulation of IGFBP5 expression and concomitant upregulation of Bcl-3, IGF1R and P-AKT levels is not only seen with MCF-7 cells, but also with BT474 cells. This suggests that the observed changes as inflicted by stromal cells are not limited to a specific cell line, but of greater importance. On the other hand, the failure of T47D cells to react this way upon exposure to stromal cells indicates that breast cancer cells do not necessarily respond to stromal cells by inducing changes in IGFBP5 and Bcl-3 expression. T47D cells expressed Bcl-3 protein at non-detectable level as judged by Western blot analysis. Hence, it is unlikely that Bcl-3 is involved in fulvestrant resistance developed by T47D cells. In agreement with this notion, recent data showed that T47D cells activate Aurora kinase B to gain fulvestrant resistance [50].

How stromal cells downregulate IGFBP5 expression in breast cancer cells has still to be determined. IGFBP5 expression has been reported to be positively regulated by STAT3 in fibroblasts [51]. However, we did not find an effect of siSTAT3 on the IGFBP5 level in MCF-7 cells (Figure 4A). Nor did siSTAT3 prevent the stromal cell-induced effects on signaling pathways and protein expression (Figure 2E). Hence, interleukin-6, the major stromal-cell secreted stimulator of STAT3 activity [52, 53], is unlikely to be responsible for IGFBP5 downregulation and its consequences. We also tested SDF-1 (stromal cell derived factor-1) and other cytokines, known to be secreted by stromal cells, for their ability to downregulate IGFBP5, but so far we did not see any effect. MSCs and CAFs also secret high amounts of PAI-1 (plasminogen activator inhibitor-1) (data not shown). This protease inhibitor has several functions, including a critical role in migration [54], which links this protein to cancer progression [55]. To analyze its importance for the stromal cell-induced effects, we eliminated PAI-1 from CAF-CM by transfecting CAFs with a PAI-1-specific siRNA, but did not find any evidence that this treatment changes the ability of CAF-CM to induce IGFBP5 downregulation in MCF-7 cells (data not shown). Besides secreted proteins, microvesicles (exosomes and shedding vesicles) as released by MSCs and CAFs [56, 57], might be responsible for the induction of IGFBP5 downregulation. In terms of shedding vesicles, we could confirm that they are released by MSCs (data not shown). We are currently studying their potential role in stromal cell-induced IGFBP5 downregulation.

By a comparative gene expression analysis of MCF-7 cells grown alone or in the presence of MSCs we identified a number of genes whose expression is downregulated in response to MSCs and CAFs. The levels of approximately half of these genes were also decreased by siIGFBP5. Of these genes, KLHL4 and SEPP1 were upregulated by siBcl3. The expression of these two genes changed along with the level of IGFBP5 in a linear fashion and inversely along with that of Bcl3. This suggests that KLHL4 and SEPP1 can be regulated through the IGFBP5/Bcl-3 axis. Interestingly, treatment of MCF-7 cells with TNFα, which stimulates NFκB activity, was found to downregulate SEPP1 [58] suggesting that SEPP1 is an NFκB/Bcl-3 target at least in MCF-7 cells. However, since downregulation of SEPP1 expression could also be induced by insulin and CoCl_2_, NFκB/Bcl-3 may act in concert with HIF1α and the PI3K/AKT pathway, all activated by stromal cell-induced IGFBP5 downregulation. One way by which the PI3K/AKT pathway could downregulate SEPP1 expression is by inhibiting the activity of the forkhead box transcription factor FoxO1A [59–61], a tumor suppressor protein shown to activate SEPP1 transcription in hepatoma cells [59]. Interestingly, in *in-silico* survival analysis, we found a correlation of higher Bcl-3 expression and lower KLHL4 and SEPP1 expression with unfavorable outcome of endocrinally treated breast cancer patients that suffered from a ERα^+^/PR^+^-tumor suggesting that not only Bcl-3 is linked to endocrine resistance, but also its target genes KLHL4 and SEPP1.

**Figure 9:**
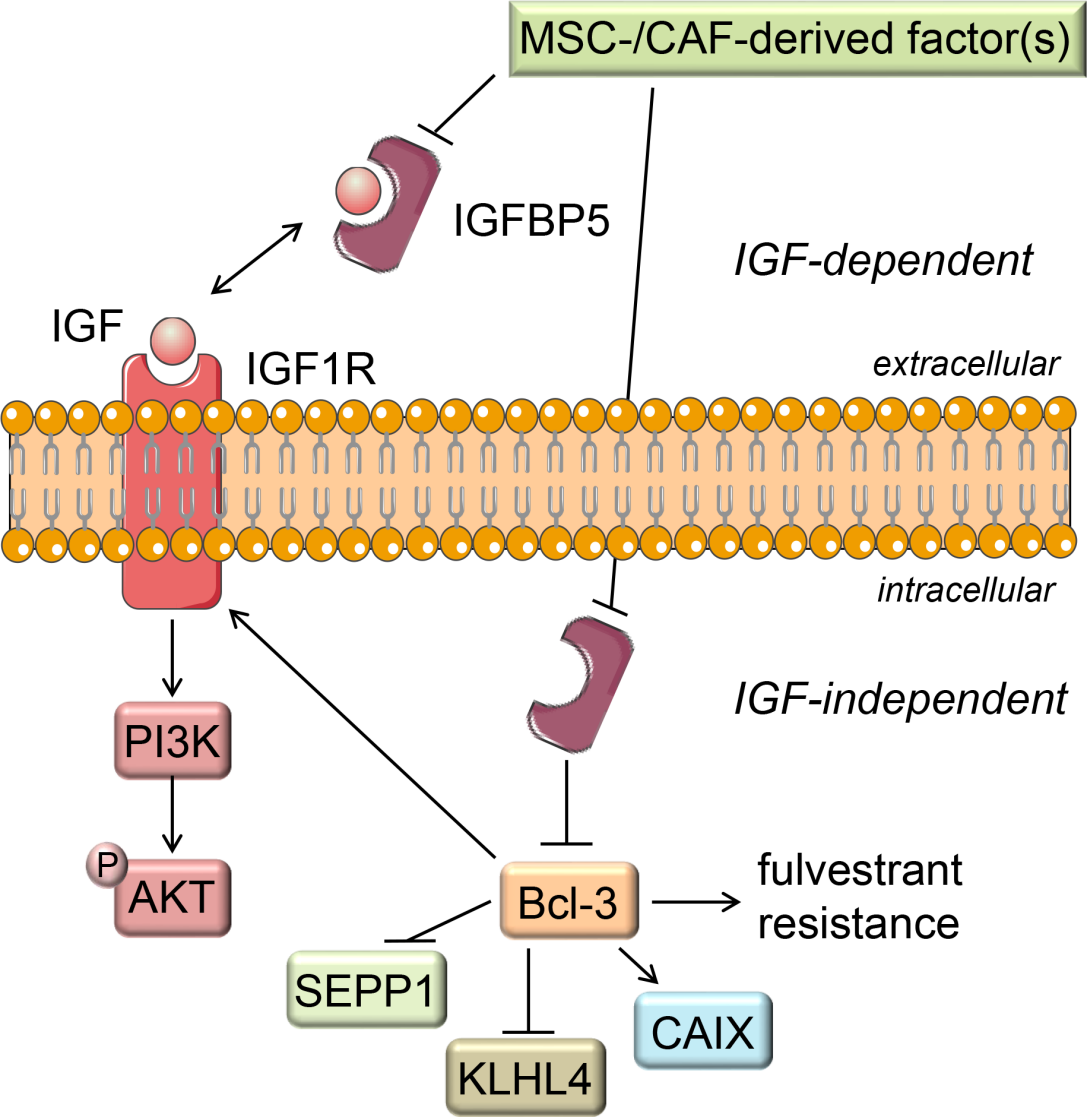
Proposed mechanism of stromal cell induced fulvestrant resistance Factor(s) secreted by MSCs and CAFs lead to a decline in the IGFBP5 expression. The reduced IGFBP5 level results in the release of IGFs from the IGF/IGFBP5 complex allowing IGF to activate IGF1R, thereby activating the PI3K/AKT signaling pathway. Besides blocking IGF activity by this IGF-dependent function, IGFBP5 also shows IGF-independent actions (e.g. as an intracellular protein). Through this IGF-independent action IGFBP5 likely keeps Bcl-3 expression down. Consequently, when stromal cells downregulate IGFBP5 expression the Bcl-3 expression raises. This leads to fulvestrant resistance and to changes in the expression of the Bcl-3 target genes IGF1R, KLHL4 and SEPP1.

In conclusion, our results suggest that MSCs and CAFs are able to trigger downregulation of IGFBP5 expression in ERα-positive breast cancer cells, such as MCF-7 cells, and, as a consequence, induce a number of changes in signaling pathway activities and gene expression (summarized in Figure 9). One of these changes, the upregulation of Bcl-3 expression, is at least partly responsible for the promoting effect of MSCs and CAFs on the cellular growth in the presence of fulvestrant. Our data also show that, even within the subgroup of ERα-positive breast cancer, cancer cells may or may not respond to MSCs and CAFs by downregulating IGFBP5 expression indicating that ERα-positive breast cancers are a heterogeneous group also in respect to their interactions with stromal cells.

Since Bcl-3 expression could be linked to unfavorable prognosis of breast cancer patients that received endocrine treatment, Bcl-3 may be a suitable biomarker for predicting endocrine therapy response of patients with ERα-/PR-positive breast cancers.

## MATERIALS AND METHODS

### Cell culture

MCF-7, BT474 and T47D breast cancer cells, whose identities were confirmed by authentication (LGC standards, Wesel, Germany or Genolytic, Leipzig, Germany), and immortalized 19TT breast CAFs [62, 63] were maintained in RPMI medium supplemented with 10% fetal calf serum (FCS, Pan Biotech) in the absence of antibiotics. Human MSCs were kindly provided by J. Lützkendorf and L. Müller. MSCs were isolated from human bone marrow and propagated as described [35]. Breast cancer cell lines and stromal cells were maintained in the same batch of serum. To obtain conditioned medium (CM) from CAFs (CAF-CM), cells were kept at 100% confluency for three days. For MSC-CM, MSCs were kept at ~50% confluency for three days (to prevent differentiation). To remove floating cells and debris, CMs were centrifuged at 3000 rpm in a Multifuge 3 (Heraeus) for 10 minutes. Unless stated otherwise, for treatment with stromal cell CM, MSC- or CAF-CM was mixed with growth medium 1+4, referred as 20% MSC-CM or 20% CAF-CM, respectively. Cells were incubated with CM for 3 days. Co-culture experiments were performed by mixing MCF-7 cells with MSCs in a ratio of 10:1 or 50:1. In transwell experiments, MSCs were separated from MCF-7 cells by a 0.4 μm pore membrane (Greiner) with the MCF-7 cells grown on the bottom of the well of a 6-well-plate and the MSCs attached to the upper side of the membrane. Spheroid assays were performed as described [35]. Based on data reported by Kirkegaard et al. [64] cell were incubated with fulvestrant at a final concentrations of 100 nM. Insulin, PQ401 and CoCl_2_ were added to cells at a final concentration of 8 μg/ml (~90 μIU/ml, as determined by insulin ELISA, Hölzel Diagnostika), 10 μM and 100 μM, respectively.

For spheroid formation in 3D suspension cultures, cells were incubated on a layer of 2% Seakem GTG agarose (dissolved in PBS) in 96-well plates at a density of 5 × 10^3^ cells/well for 3–4 days in the presence of fulvestrant and/or CAF-CM or in the absence of both agents. To measure the spheroid size, a picture was taken by an AxioCam MRc 5 camera and the area displayed on this picture measured by AxioVision R 4.5 software as described previously [65].

### Antibodies and reagents

For Western blot analysis, the following antibodies were used (working dilutions are given in brackets). Rabbit polyclonal antibodies: anti-P(S473)-AKT (1:2000, D9E, Cell Signaling), anti-Bcl-3 (1:1000, C-14, Santa Cruz), anti-P(Thr202, Tyr204)-ERK1/2 and anti-ERK1/2 (both 1:2000, Cell Signaling), anti-ERα (1:2000, Santa Cruz, HC-20), anti-IGF1Rβ (1:2000, Cell Signaling), anti-P(Tyr705)-STAT3 (1:1000, D3A7, Cell Signaling) and anti-STAT3 (1:1000, 79D7, Cell Signaling); rabbit monoclonal antibodies: anti-integrin β1 (1:2000, EPR1040Y, Abcam), anti-GAPDH (1:5000, Ambion) and Ki67 (1: 2000, Epitomics, clone EPR3610); mouse monoclonal antibodies: anti-(pan)AKT (1:1000, 40D4, Cell Signaling), anti-E-cadherin (1:5000, BD Transduction Lab.) and anti-HIF1α (1:1000, BD Transduction Lab.). Anti-CAIX was kindly provided by S. Pastorekova. Secondary antibody conjugates (anti-rabbit/anti-mouse horse radish peroxidase, 1:2000) were purchased from Cell Signaling.

Fulvestrant (LKT Laboratories) was purchased from Biomol (Hamburg/Germany), PQ404 from Calbiochem and recombinant human insulin was from Sigma-Aldrich.

### RNA interference

Small interference (si)RNAs were purchased from Eurofins MWG. Transfection was performed by electroporation as described [66]. Briefly, cells were trypsinized, washed once in RPMI medium, electroporated by using a Bio-Rad GenePulserX-Cell at 250 V and 800 μF and kept on ice for 30 min. Cells were then transferred to a 6 or 10 cm (Ø) culture dish and incubated for two days to allow the siRNA to downregulate the expression of its target. The effect of the siRNA was confirmed by Western blot and/or Q-RT-PCR analysis. The following siRNAs (sense-strand) were used: siBcl3 (5′-UGG UCU UCU CUC CGC AUC A-3′), siLuc (5′-CUU ACG CUG AGU ACU UCG A-3′), siIGFBP5 (5′-GCA GAU CUG UGA AUA UGA A-3′) and siSTAT3 (5′-GAA UCA CGC CUU CUA CAG A-3′).

### Growth assays

To determine cell growth of individual clones, cells were trypsinized, counted and seeded on a 10 cm (Ø) petri dish (3 × 10^4^ cells per dish) in 10 ml growth medium. Cells were then incubated with MSC-CM, CAF-CM or insulin and/or fulvestrant or left untreated for five days. Cell growth of individual clones were determined by measuring the size of each clone by using an AxioCAM MRc5 camera and the AxioVision R 4.5 imaging software (Zeiss). Single cells were not counted. For each condition, at least fifty individual clones were randomly chosen and measured. In RNA interference experiments, cells were transfected with siRNA and incubated for two days before the clonogenic assay was started.

To examine cell growth activity at higher cell density an ATP-based assay (Vialight Plus Kit, Lonza) was used. Cells were seeded at a density of 1 × 10^4^ or 3 × 10^4^ per well of a 24-well plate and incubated for 5 to 7 days in the presence of fulvestrant and/or CAF-CM or in the absence of both. After removal of the growth medium, cells were washed once with PBS and lysed by adding a mixture of 100 μl PBS and 50 μl lysis buffer. Cell lysates were cleared by microfugation at 4000 rpm for 5 min. After 75 μl of the cleared lysate was mixed with 50 μl luciferase stock solution, the mixture was incubated for 2 min at RT and luciferase activity measured in a Sirius luminometer (Berthold).

### Quantitative RT-PCR

RNA isolation, cDNA synthesis and quantitative (Q) PCR were carried out as described [66], except that the RNA isolation kit was from Roche and the dNTP mix was from Qiagen. Briefly, cDNA synthesis was done by using Superscript II (Invitrogen) by starting from 1 μg total RNA. For Q-PCR, ABsolute QPCR SYBR Green Fluorescein Mix (Thermo Fisher Scientific Biosciences) was used. PCRs were run in a BioRAD iCycler. Results were analyzed by iQ5 Optical System software version 2.1. Relative RNA levels of genes were calculated by the comparative *Ct* (2^−ΔΔ*Ct*^) method by using GAPDH and HPRT as reference genes for normalization. The primers used for Q-PCR are listed in Supplementary Table S1.

### cRNA microarray analysis

In two independent experiments, 5 × 10^5^ MCF-7 cells were seeded into the well of a 6-well plate alone or together with 1 × 10^4^ MSCs and grown for two days before total RNA was isolated. After the RNA was quality-checked by an Agilent 2100 Bioanalyzer, gene expression analyses were performed by Miltenyi Biotec by using Agilent Whole Human Genome Oligo Microarrays 8 × 60K. Briefly, cRNAs were generated from 100 ng of each RNA sample by the Agilent Low Input Quick AMP labeling kit (Agilent Technologies). RNAs from control samples (MCF-7 alone) were labeled with Cy3, those from MCF-7/MSC co-culture samples with Cy5. Of the corresponding Cy3- and Cy5-labeled fragmented cRNAs, 300 ng each were combined and hybridized o/n to the oligo microarray by following the instructions of the manufacturer (Agilent Technologies). Imaging and data analysis was carried out by using Agilent Feature Extraction Software.

### Protein extraction and western blot analysis

Protein extractions from the membraneous, cytosolic and nuclear fractions and Western blot analysis were carried out as described [66]. Briefly, after having been scraped off the plate, cells were centrifuged and resuspended in 400 μl buffer A (10 mM HEPES (pH 7.9), 10 mM KCl, 0.1 mM EDTA, 0.1 mM EGTA) and passed through a 20-gauge needle. Stepwise centrifugation at 3000, 6500 and 13000 rpm in a microfuge was used to obtain cytosolic, nuclear and membraneous protein fractions. For nuclear or membraneous protein extraction, the pellet was extracted in buffer C (20 mM HEPES (pH 7.9), 400 mM NaCl, 1 mM EDTA, 1 mM EGTA, 1 mM DTT) or buffer D (5 mM HEPES (pH 7.9), 0.5 mM K-EDTA (pH 7.2), 1 mM DTT), respectively. For HIF1α detection, whole cell extracts (WCE) were prepared as described [67]. Briefly, depending on cell density, cell layers were incubated with 100–300 μl ice-cold RIPA lysis buffer (50 mM Tris HCl (pH 7.4), 200 mM NaCl, 1 mM EDTA, 1 mM EGTA, 1% TritonX-100, 0.25% deoxycholate) containing a protease inhibitor cocktail (1:100) (Sigma-Aldrich, P8340) for 20 min and scraped off from the plate. Lysates were cleared by microfugation at full speed for 10 min.

Ten μg protein of each sample was separated on a 10% SDS-polyacrylamide gel and transferred to a PVDF membrane (Millipore). After blocking the membrane in 2% skim milk (Applichem) dissolved in washing buffer (10 mM Tris/HCl (pH 7.5), 100 mM NaCl, 1 mM EDTA), it was sequentially incubated with the primary antibody and the secondary antibody in washing buffer containing 0.2% skim milk. Peroxidase activity was visualized by chemoluminescence using ECLPlus and Hyperfilm ECL (GE Healthcare).

### Statistical analyses

Data obtained from colony growth assays were analyzed by Wilcoxon matched pair test. For other two group comparisons, two sample *t*-test or paired *t*-test were used depending on whether the data were dependent or independent. A *p* value of *p* < 0.05 was considered to be statistically significant. For all graphs, *p < 0.05, **p < 0.01, ***p < 0.001, ****p < 0.0005, *****p < 0.0001.

## SUPPLEMENTARY FIGURE AND TABLES




