# Supplementary material for: Stromal cells promote anti-estrogen resistance of breast cancer cells through an insulin-like growth factor binding protein 5 (IGFBP5)/B-cell leukemia/lymphoma 3 (Bcl-3) axis
Source: Oncotarget. 2015 Oct 19;6(36):39307–28. doi: 10.18632/oncotarget.5624 (PMC4770774; doi:10.18632/oncotarget.5624)
Supplement: Supplementary file 1 [file oncotarget-06-39307-s001.pdf]

SUPPLEMENTARY FIGURE AND TABLES

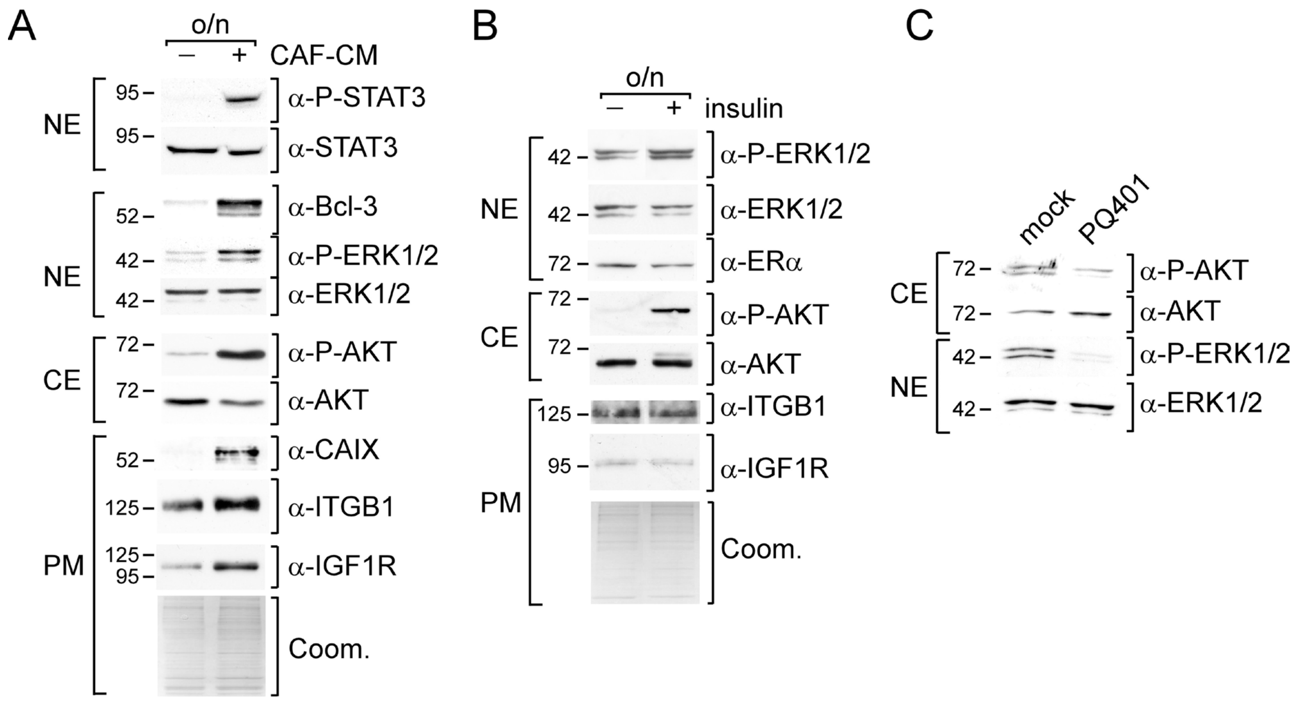

Supplementary Figure S1: Western blot analyses of proteins and phospho-proteins as indicated. Overnight incubation of MCF-7 cells with 20% CAF-CM **A.** insulin **B.** or IGF1R inhibitor PQ401 **C.** or none of these agents. CE, NE, PM = cytosolic, nuclear and plasma membrane extract. Coom. = Coomassie Blue stained.

Supplementary Table S1: Primers for Q-PCR

Gene	Forward primer (5′- > 3′)	Reverse primer (5′- > 3′)
ABCG2	TGCAACATGTACTGGCGAAGA	GAGAGATCGATGCCCTGCTT
ACTA2	GAGTTGCCTGATGGGCAAGT	GGCAGCGGAAACGTTCATT
ALDH3A1	GTCCCTGAGACCACGGAGC	CCCGTGTACAGGATATGGTCG
BCL3	GAAGCACATGCACCTACCCA	GGACTGGTGCCTGAGAATGC
CD44	CCATTTTGCCCTTCCATAGC	CAACCCCCAACCTCAGTGG
FGF18	ACACTTCCTGCTGCTGTGCTT	GAAGTCCACGTTCTCCTCGG
FN1	TGCGAGAGTAAACCTGAAGCTG	ACCCACTCGGTAAGTGTTCCC
GAPDH	GAAGGTGAAGGTCGGAGT	GAAGATGGTGATGGGATTTC
HPRT	GGACAGGACTGAACGTCTTGC	TGAGCACACAGAGGGCTACAA
IGF-1	CTTCTACCTGGCGCTGTGC	CCATACCCTGTGGGCTTGTT
IGF-2	GACGTA CTGTGCTACCCCCG	TTGGAAGAACTTGCCCACG
IGFBP1	ATTTCCATCTGATGGCCCC	CGTCCCAAAGGATGGAATGAT
IGFBP2	GGTTGCAGACAATGGCGAT	TGGTTCTCCACCAGGCCTC
IGFBP3	CAGCGCTACAAAGTTGACTACGA	GAAGTTCTGGGTATCTGTGCTCTG
IGFBP4	AGCACTTCGCCAAAATTCGA	TGACCTTCATCTTGCCCCC
IGFBP5	CCAATTGTGACCGCAAAGG	CGGGAAGGTTTGCACTGCT
IGFBP6	CATGGAGCTGTCATCACTCAACA	TGAAGGTGGATTGAGGGCC
IGFBP7	AACTGGCTGGGTGCTGGTATC	TATTCTCCAGCATCTTCCTTACTTAGAG
KIF12	CCCTTCGCGCCTCTTATCT	GCAAGTCCCGAACCTGCTC
KLHL4	TGATTGCAGGACACCTCCG	CTGCGCTGAGAACCAACCTAT
KLK11	AGCCCCGCTACATAGTTCACC	CAGCCCTCCTCCTTCTGGA
PROCR	CGATACTGCTGCTGTCTGGC	CATCTGAGGCGTCTTGGCTAC
RAB30	CCTGTGAGGAATCCTTCCGTT	GTTCTATCTCCCGCAGCCACT
RAMP3	CATGATGGGCAAGGTGGAC	ATGAACTCGGACAGGTTGCAC
SEPP1	CAGGCCTTCATCACCACCAT	GGTGACCCTGCCTATGCTGA
TGFBR3	GGCTGTGCAGGAAGAAGCTATC	TTCCTTGTGTGTGCTGGCAT
TMEM26	GAGGGTTGCATCAGCTCCA	CGACTCCCGTCACTCAACAAG
UGT2B15	GGTCATCCCAAAACCAAAGCT	GATGCCATTGGTTCCACCAT
VIM	GCAGGAGGCAGAAGAATGGTA	CAGCCTCAGAGAGGTCAGCAA
YPEL1	ACACGCCCTCCTGGACAAC	AGCAACACTGGAAAATGCACG

Supplementary Table S2: Differentially expressed genes (fold change ≥ 2.0) in MCF-7/MSC co-cultures (50:1) vs. MCF-7 cultures.
